# Spinal Distortion

**Published:** 1844-10-19

**Authors:** 


#### SPINAL DISTORTION.

Two cases of extreme spinal distortion are related by Mr. Hare in the Provincial Medical Journal, in both of which a cure was effected by placing the patients on an inclined plane, making gentle extension by weights attached to the feet, axilla, and head, and by attention to the general health. The first case is described as caries of the dorsal vertebræ, but a careful perusal of the history of the case has convinced us that it is a simple case of angular distortion only.—*Ibid.*

